# Combined endoscopic and robotic-assisted transcolonic polypectomy as a novel technique for challenging polyp resection

**DOI:** 10.1007/s00464-025-12445-2

**Published:** 2025-12-08

**Authors:** Emily Nghiem, Sophia Zigouras, Hongyan Yu, Christian Adkisson, Othon Wiltz, James Taylor

**Affiliations:** 1 Department of Surgery, Montefiore Medical Center, Bronx, NY USA; 2 Department of Surgery, Division of Colorectal Surgery, Montefiore Medical Center, Bronx, NY USA; 3 Department of Surgery, Albert Einstein College of Medicine, 182 East 210th Street - (Lower Level), Bronx, NY USA; 4 Department of Surgery, Division of Colorectal Surgery, Rutgers Robert Wood Johnson University Hospital, New Brunswick, NJ USA; 5 Department of Cardiothoracic Surgery, Baylor Scott & White Health, Plano, TX USA

**Keywords:** Colorectal cancer, Combined endoscopic-laparoscopic surgery, Combined endoscopic-robotic surgery, Robotic-assisted polypectomy, Transcolonic polypectomy, Minimally invasive surgery

## Abstract

**Background:**

Colonoscopic resection of adenomatous polyps reduces mortality from colorectal cancer, which remains the third leading cause of cancer-related deaths in the U.S. Most polyps can be removed endoscopically by cold snare resection, endoscopic mucosal resection, or endoscopic submucosal dissection. However, technically challenging polyps often require partial colectomy, which confers increased rates of morbidity and mortality. Emerging hybrid approaches such as combined endoscopic-laparoscopic techniques have been developed to manage benign, complex colonic polyps and reduce the need for surgical resection.

**Objective:**

This paper describes a novel technique of combined endoscopic and robotic-assisted transcolonic polypectomy for the management of a benign, complex colonic polyp not amenable to conventional endoscopic resection.

**Methods:**

A hybrid approach integrating endoscopic visualization via colonoscope with robotic-assisted transcolonic access was performed to achieve complete polypectomy in a patient with a technically challenging, benign colonic lesion and a past surgical history of extended right colectomy. The technique was evaluated in terms of feasibility, adequacy of resection, and operative considerations.

**Results:**

The combined endoscopic and robotic-assisted approach successfully enabled complete resection of a benign, complex colonic polyp that had previously failed endoscopic submucosal dissection. The procedure was performed without significant complications and allowed for reduced operative time and anticipated shorter recovery as compared to partial colectomy.

**Conclusions:**

This case demonstrates that combined endoscopic and robotic-assisted transcolonic polypectomy is a safe, feasible, minimally invasive alternative for select complex, benign colonic polyps. This novel technique may reduce the need for partial colectomy and associated morbidity, offering a promising option for managing challenging lesions, especially in patients with multiple comorbidities and high operative risk.

**Supplementary Information:**

The online version contains supplementary material available at 10.1007/s00464-025-12445-2.

Although the mortality rate of colorectal cancer has dropped by over 50% over the past 5 decades, largely in part due to increased awareness and screening efforts, it is still the third leading cause of cancer-related deaths in the U.S [1]. 15–30% of cases present with metastasis upon diagnosis, with 20–50% of initially localized cases eventually progressing to metastasis [2]. Meanwhile, the 5-year survival rate in the setting of distant disease remains low at around 15%, highlighting the importance of colorectal cancer screening for early detection [3]. Colonoscopic resection of adenomatous polyps has been shown to reduce mortality from colorectal cancer [4]. While small, sub-centimeter lesions are usually amenable to cold snare resection, larger or non-pedunculated lesions may require more involved procedures such as endoscopic mucosal resection (EMR) or endoscopic submucosal dissection (ESD). However, some polyps may still pose technical challenges in the setting of endoscopic resection, with partial colectomy as the next-line standard of care. Given the higher rates of morbidity and mortality associated with the more invasive nature of surgical resection, over the past few decades much work has gone into developing hybrid techniques combining endoscopy and minimally invasive surgery with the goal of minimizing risk while still performing adequate resection. Combined endoscopic–laparoscopic surgery (CELS) has been shown to effectively treat benign, complex colonic polyps with shorter operating time, lower morbidity, and shorter hospital stay when compared to standard colonic resection [5]. CELS has also proven to be a successful alternative to colonic resection in the treatment of early-stage colon cancer in patients with low risk of lymph node metastasis and high operative risk [6]. Here, we present a case combining endoscopy with robotic-assisted transcolonic polypectomy.

## Case presentation

**Fig. 1:**
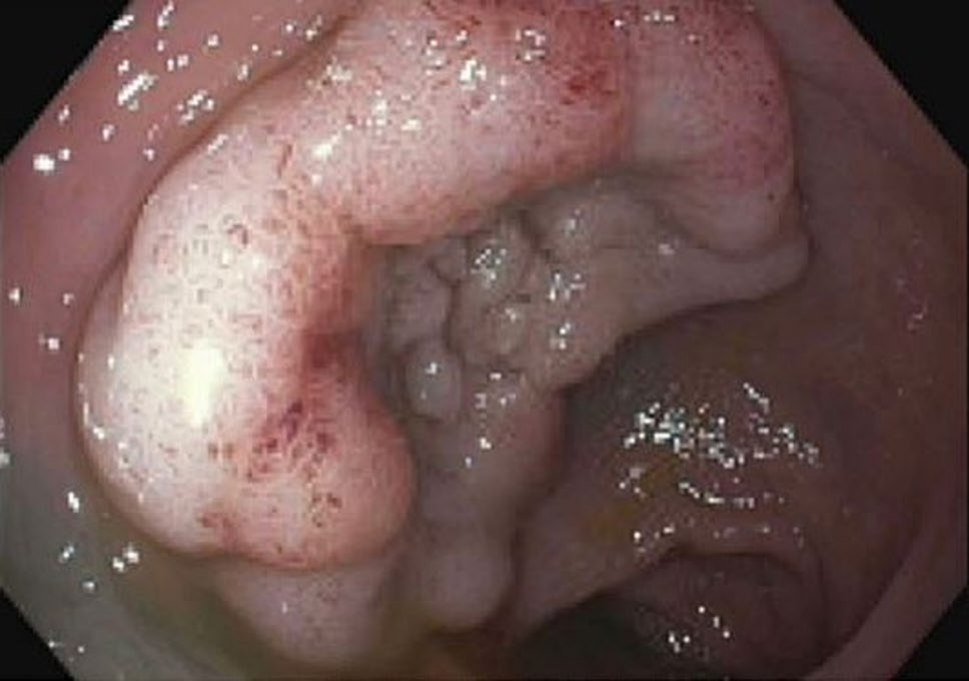
A large (> 2 cm) tubulovillous adenomatous polyp is seen in the sigmoid colon during colonoscopy (Color figure online)

**Fig. 2:**
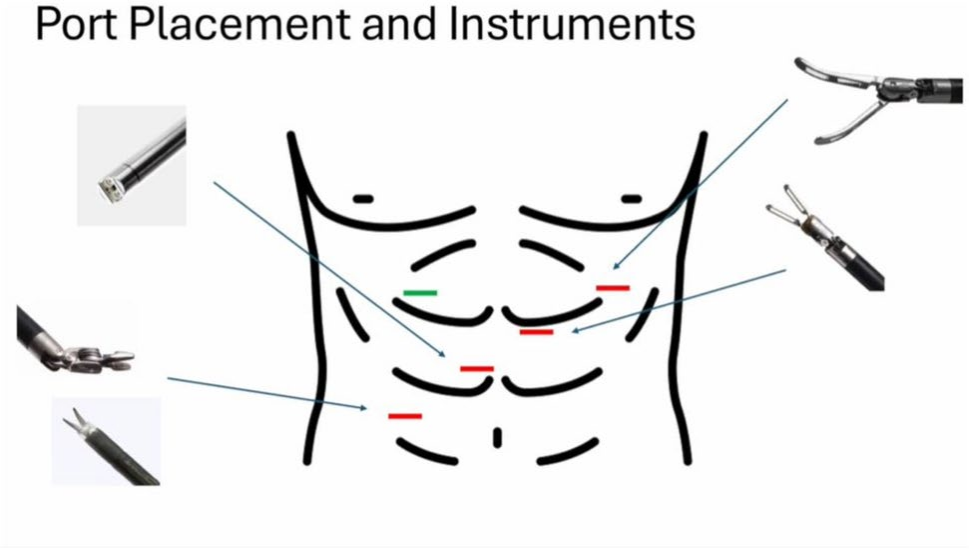
Placement of Da Vinci X robotic 8 mm ports is indicated in red along with the corresponding instruments used; AirSeal placement is indicated in green (Color figure online)

**Fig. 3:**
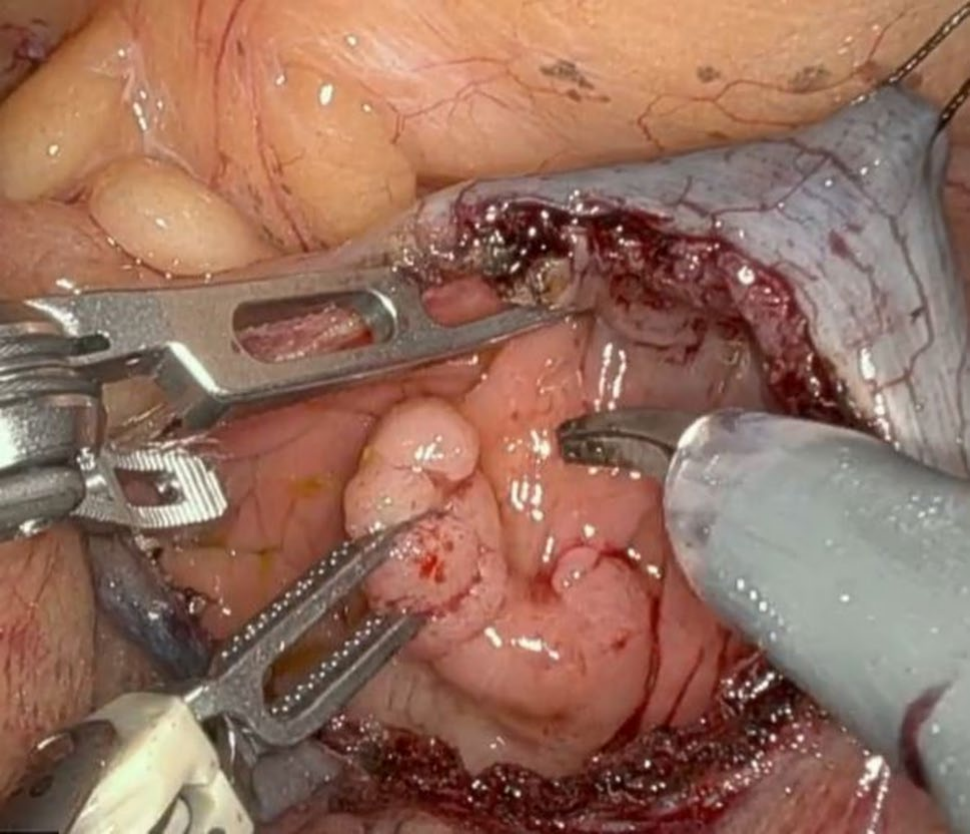
A 3–0 silk stay suture placed superiorly to the colotomy is grasped through the abdominal wall to tent up the colon, allowing for exposure and manipulation of the polyp (Color figure online)

**Fig. 4:**
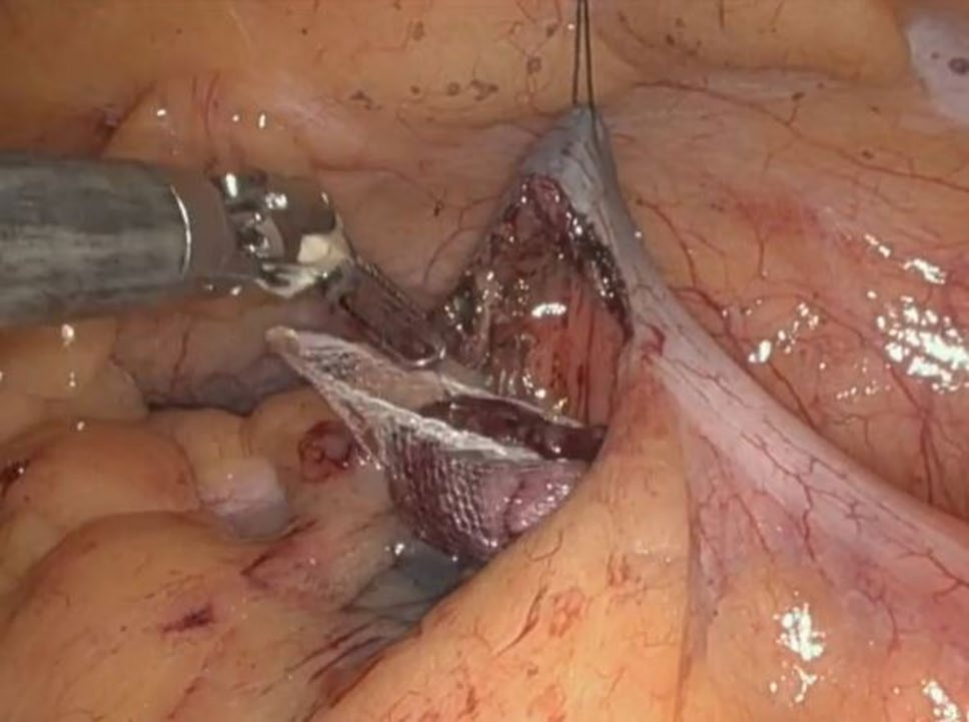
Following resection, the polyp is removed in an endo-catch bag via the colonoscope (Color figure online)

**Fig. 5:**
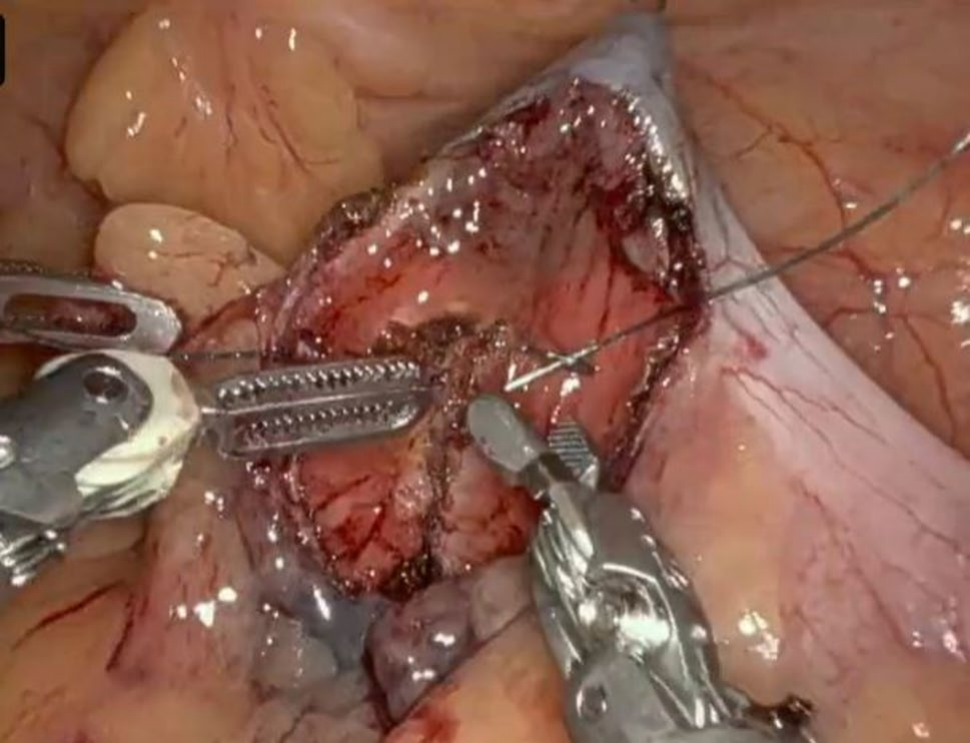
Transverse closure of the resection bed using a 3–0 V-lock suture (Color figure online)

A 62-year-old male with a past surgical history of extended right colectomy for pT3bN1 MSS transverse colon cancer presented for surveillance colonoscopy 8 years postoperatively, with prior colonoscopies at postoperative years 2 and 5 showing no evidence of disease. The most recent colonoscopy showed evidence of a large (> 2 cm) polyp in the sigmoid colon, with biopsies demonstrating tubulovillous adenoma (Fig. 1). Endoscopic submucosal resection was initially attempted; however, the lesion was unable to be lifted. The area was tattooed, and the procedure was subsequently aborted. Surgical options included completion colectomy with ileorectal anastomosis, as the inferior mesenteric artery was the only remaining vessel supplying the colon, or an attempt at a transcolonic polypectomy, with the knowledge that any evidence of cancer would necessitate a future oncologic resection. Due to the patient’s prior partial colectomy, there was concern for lack of appropriate perfusion and lack of adequate reach if completion colectomy were to be performed. In light of this, the decision was made to proceed with transcolonic polypectomy with endoscopic retrieval of the polyp, in order to avoid further colonic resection. Four 8 mm ports and an AirSeal were placed, and the Da Vinci X robot was docked while, concurrently, colonoscopy was performed (Fig. 2). After lysis of adhesions, the previously placed tattoo was identified on the anti-mesenteric side of the colon, opposite to the lesion, and a longitudinal colotomy was created to allow access to the polyp. A 3–0 silk stay suture was placed superior to the colotomy and grasped through the abdominal wall to tent up the colon (Fig. 3). Robotic scissors were used to mark the planned resection margin, and the dissection was carried out below the submucosal plane. Once fully resected, the polyp was removed in an endo-catch bag via the colonoscope (Fig. 4). The resection bed was closed transversely with a 3–0 V-lock suture (Fig. 5). The colotomy was then closed transversely in two layers – first using a running 3–0 V-lock suture and then using a 3–0 V-lock Lembert suture to imbricate the suture line. Throughout closure, the colonoscope was used to visualize the colon lumen, ensuring no narrowing. Upon completion, the colonoscope was advanced past the area of closure to ensure patency and rule out any gross leaks. The patient was discharged 24 h postoperatively after return of bowel function and tolerating diet. Final pathology demonstrated a 2.6 × 2.5 × 0.9 cm adenoma with focal villous features and the cauterized margins were free of adenoma.

## Discussion

Given the increasing emphasis on prevention and early detection, endoscopic resection of adenomatous polyps has become a mainstay of colorectal cancer screening. However, up to 10–15% of polyps prove too technically challenging for colonoscopic resection, necessitating partial colectomy as the next-line treatment [7]. There is a need for novel interventions as surgical resection can confer complication rates of up to 35%, including anastomotic leak rates of up to19% and mortality rates of up to 4% [8, 9]. Ideally, less invasive measures could be taken to treat these benign yet potentially pre-malignant lesions.

In this paper, we describe a novel technique combining endoscopy with robotic-assisted transcolonic polypectomy. Though no data on the efficacy and safety of this exact technique currently exist in the literature, similar minimally invasive techniques have been studied. A 2021 study out of the Netherlands compared laparoscopic wedge resection (LWR) for benign endoscopically unresectable polyps to oncological colon resection (OCR), examining clinical and histological outcomes. The LWR group experienced a lower rate of major complications at 4%, compared to 16.7% in the OCR group. The LWR group also had a significantly shorter median length of hospital stay of 2 days, compared to 5 days for the OCR group [8]. The results of this study support the less invasive laparoscopic wedge resection as a safe and effective alternative treatment for endoscopically unresectable benign colonic polyps. Marres et al. notably draw an important comparison – while the rate of major complication in the OCR group was comparable to that of the total national population undergoing oncologic colon surgery, these numbers are less acceptable in the setting of benign lesions. While most data on minimally invasive techniques for polypectomy focus on benign pathology, the 2023 study examining CELS for the treatment of early colon cancer also supports the application of less invasive, lower-risk techniques in the setting of oncologic resection. In this study, 20 out of 22 high-risk colon cancer patients achieved free resection margins with a median hospital stay of 1 day. Over 33% of colon cancer patients have a functional performance status that confers a 1-year mortality risk of up to 18% after elective colon cancer surgery [6]. In light of this, new advancements in minimally invasive techniques such as the one described in this paper may further the treatment of both benign and malignant disease.

**Fig. 6:**
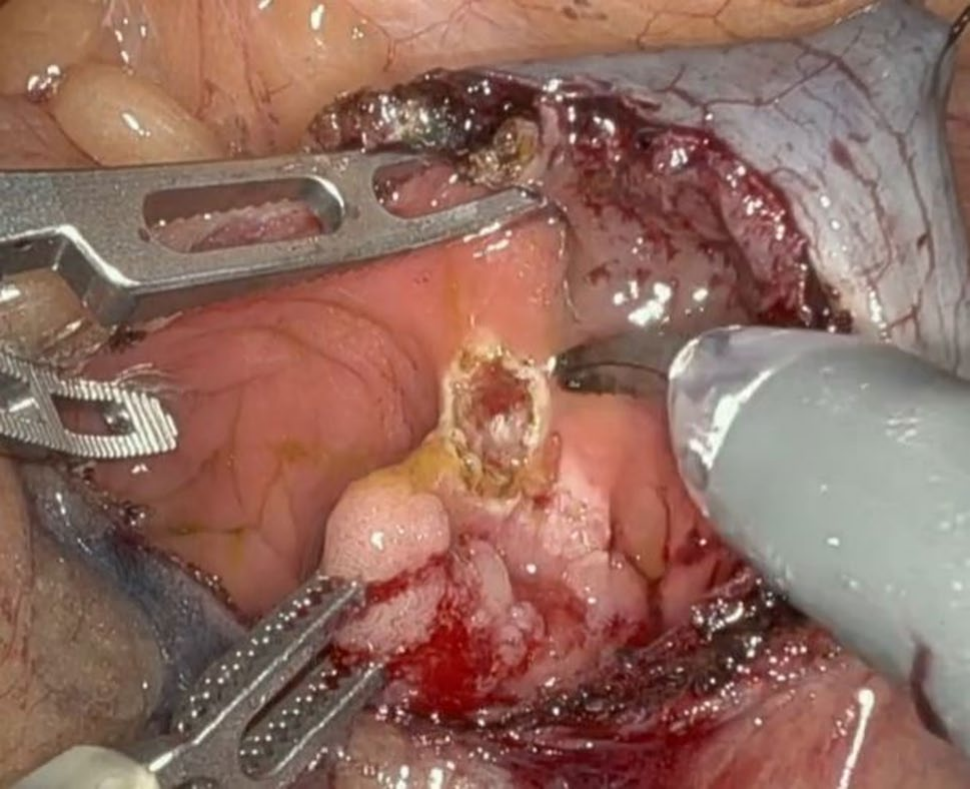
The use of robotic instruments allows for better grasping and retraction of the polyp, which was not possible during initial attempts at endoscopic submucosal dissection (Color figure online)

Robotic-assisted transcolonic polypectomy harbors several technical advantages. As previously mentioned, the main advantage is the lack of colonic resection, forgoing the need for anastomosis and its associated complications, namely anastomotic leak. It is also advantageous in comparison with similar minimally invasive techniques. It uses the same submucosal dissection as that of ESD; however, the robotic component allows the polyp to be repeatedly grasped and adjusted through the colotomy allowing for a better ability to lift the lesion, which can be a limiting factor in ESD (Fig. 6). When compared to LWR, which involves a full thickness en bloc resection of the polyp, transcolonic polypectomy is more readily applicable to mesenteric polyps, as described in the case presentation. However, in the case of an anti-mesenteric polyp, a colotomy could also be made at the anti-mesenteric border, proximal or distal to the polyp, with the incision guided by the colonoscope to ensure appropriate margins. The transcolonic aspect of this technique avoids the larger trochars needed to accommodate the staplers used for bowel anastomosis, therefore avoiding the increased pain associated with larger port sites. Meanwhile, the colonoscopic aspect of this technique also obviates the need for an extraction site, as the specimen is removed endoscopically. This avoids the increased risk of port site hernia and surgical site infection associated with larger incisions, especially those used for specimen extraction [10]. One main limitation of robotic-assisted transcolonic polypectomy is tumor location. Tumors near the hepatic or splenic flexure would require more involved dissection, such as taking down the line of Toldt, to allow for adequate mobility when grasping the tumor through the colotomy. This technique is best suited for colonic tumors in the lower pelvis requiring less dissection for exposure.

## Conclusion

With various minimally invasive techniques to approach endoscopically unresectable colonic polyps emerging, we describe a novel method of robotic-assisted transcolonic polypectomy. It is comparable to laparoscopic wedge resection, which has been studied and determined to have lower rates of major complications and a shorter length of hospital stay when compared to oncologic colonic resection. As with any procedure, patient selection remains a key factor – this technique lends itself well to those with multiple comorbidities who pose a high operative risk, which is not uncommon in the population of patients undergoing colorectal cancer screening. In the case of our older patient with a prior oncologic partial colectomy, this colon-sparing method conferred lower risk while still providing a successful outcome. However, with its promising results and safety profile, if more widely implemented in practice, this technique could serve as a less invasive treatment option for patients of all populations.

## Supplementary Information

Below is the link to the electronic supplementary material.

Supplementary file1 (MP4 168389 KB)
