# Correction: Combined endoscopic and robotic-assisted transcolonic polypectomy as a novel technique for challenging polyp resection

**DOI:** 10.1007/s00464-025-12485-8

**Published:** 2025-12-15

**Authors:** Emily Nghiem, Sophia Zigouras, Hongyan Yu, Christian Adkisson, Othon Wiltz, James Taylor

**Affiliations:** 1 Department of Surgery, Montefiore Medical Center, Bronx, NY USA; 2 Department of Surgery, Division of Colorectal Surgery, Montefiore Medical Center, Bronx, NY USA; 3 Department of Surgery, Albert Einstein College of Medicine, 182 East 210th Street - (Lower Level), Bronx, NY USA; 4 Department of Surgery, Division of Colorectal Surgery, Rutgers Robert Wood Johnson University Hospital, New Brunswick, NJ USA; 5 Department of Cardiothoracic Surgery, Baylor Scott & White Health, Plano, TX USA

**Correction to: Surgical Endoscopy** 10.1007/s00464-025-12445-2

The original online version of this article was revised to correct the abstract text. The full, correct abstract for this article is as follows:

Background: Colonoscopic resection of adenomatous polyps reduces mortality from colorectal cancer, which remains the third leading cause of cancer-related deaths in the U.S. Most polyps can be removed endoscopically by cold snare resection, endoscopic mucosal resection, or endoscopic submucosal dissection. However, technically challenging polyps often require partial colectomy, which confers increased rates of morbidity and mortality. Emerging hybrid approaches such as combined endoscopic-laparoscopic techniques have been developed to manage benign, complex colonic polyps and reduce the need for surgical resection.

Objective: This paper describes a novel technique of combined endoscopic and robotic-assisted transcolonic polypectomy for the management of a benign, complex colonic polyp not amenable to conventional endoscopic resection.

Methods: A hybrid approach integrating endoscopic visualization via colonoscope with robotic-assisted transcolonic access was performed to achieve complete polypectomy in a patient with a technically challenging, benign colonic lesion and a past surgical history of extended right colectomy. The technique was evaluated in terms of feasibility, adequacy of resection, and operative considerations.

Results: The combined endoscopic and robotic-assisted approach successfully enabled complete resection of a benign, complex colonic polyp that had previously failed endoscopic submucosal dissection. The procedure was performed without significant complications and allowed for reduced operative time and anticipated shorter recovery as compared to partial colectomy.

Conclusions: This case demonstrates that combined endoscopic and robotic-assisted transcolonic polypectomy is a safe, feasible, minimally invasive alternative for select complex, benign colonic polyps. This novel technique may reduce the need for partial colectomy and associated morbidity, offering a promising option for managing challenging lesions, especially in patients with multiple comorbidities and high operative risk.

The original article has been corrected.

